# Comorbidity and diagnosis distribution in transdiagnostic treatments for emotional disorders: A systematic review of randomized controlled trials

**DOI:** 10.1371/journal.pone.0207396

**Published:** 2018-11-15

**Authors:** Alberto González-Robles, Amanda Díaz-García, Clara Miguel, Azucena García-Palacios, Cristina Botella

**Affiliations:** 1 Department of Basic and Clinical Psychology, and Psychobiology, Universitat Jaume I, Castellon, Spain; 2 CIBER Fisiopatología Obesidad y Nutrición (CIBERObn), Instituto Salud Carlos III, Madrid, Spain; University of California Los Angeles, UNITED STATES

## Abstract

The advantages of transdiagnostic protocols for emotional disorders (ED) (anxiety and depression) include the ability to treat multiple psychological disorders using the same treatment protocol, and the capacity to better address comorbidity. Comorbidity in ED has been associated with higher rates of severity, functional impairment, and chronicity. However, no attempts have been made in the literature to systematically review whether these studies include assessments to evaluate the treatment response in comorbid diagnoses, in addition to the principal diagnosis. Moreover, transdiagnostic treatments have been developed for a range of ED, but to date no study has analyzed the real distribution of diagnoses in these studies. The current study aimed to analyze: a) whether treatment response in comorbidity is evaluated in transdiagnostic treatments for ED; b) what diagnoses are targeted in transdiagnostic treatments for ED; and c) the real distribution of the diagnoses at baseline in these studies. A systematic search of the literature was conducted in PsycINFO, PubMed, EMBASE, and the Cochrane Library. Fifty-two randomized controlled trials were identified, with a total of 7007 adult participants. The results showed that, although most of the studies reported data on comorbidity at baseline, only 40% of them examined the effects of the intervention on the comorbid disorders. The most commonly targeted diagnoses in transdiagnostic protocols were panic/agoraphobia, generalized anxiety, social anxiety, and depression. Other disorders, such as obsessive-compulsive disorder, posttraumatic stress disorder, and anxiety/depression not otherwise specified, were marginally included in these studies. Regarding the distribution of diagnoses at baseline, generalized anxiety, panic/agoraphobia, social anxiety, and depression were the most frequently observed, whereas depression not otherwise specified was the least represented. The results highlight the importance of assessing comorbidity in addition to the principal diagnoses in transdiagnostic treatments, in order to draw conclusions about the true potential of these interventions to improve comorbid symptoms. Implications of the current study and directions for future research are discussed.

## Introduction

Emotional disorders (ED) (depression and anxiety disorders) are common mental health conditions and one of the main causes of suffering and impairment worldwide [1,2]. In the past few decades, a large number of disorder-specific cognitive-behavioral treatments (CBT) have been developed for ED and tested in clinical trials, with evidence found for their efficacy and effectiveness [3–7]. However, although disorder-specific treatment protocols have been shown to work effectively, there are still some barriers related to these protocols. One of them stems from the high comorbidity rates observed in ED, ranging between 40 and 80% for these disorders [8, 9].

Comorbidity in ED has been associated with greater severity and impairment [8], worse quality of life [10], and higher chronicity rates [11]. The literature has proposed different ways to manage comorbidity, such as combinations of treatments or the sequential application of treatments [12]. Another strategy involves applying a protocol to target one of the disorders and expecting an impact on the comorbid disorders. Nevertheless, the effective use of these strategies is not well supported by the existing empirical evidence (for a review of the evidence, see McManus et al., 2010) [12]. A more recent development to deal with comorbidity is the application of treatments based on a transdiagnostic perspective. Although the term *transdiagnostic* has been employed to refer to different treatment approaches [13], the common denominator of these treatments is that one protocol is applied to address various psychological disorders [14].

Research on transdiagnostic treatments for ED has increased in recent years [15–17], with a noteworthy rise in the number of trials assessing the efficacy and effectiveness of transdiagnostic treatments in the past 15 years [18–27]. Several advantages have been attributed to transdiagnostic treatments. The first and most important is the ability to address multiple ED using the same treatment protocol. Thus, these disorders can be treated in a more cost-effective way because clinicians only have to be trained in one protocol that addresses various psychological disorders [13, 15]. Second, training clinicians in one treatment approach, rather than in a different protocol for each ED, may facilitate the dissemination of evidence-based treatments for these specific problems [15]. This approach could be of particular interest in ecological settings such as public services, where clinicians have to treat patients with diagnostically heterogeneous presentations, which makes the adequate selection of protocols and techniques difficult [13]. Third, another important advantage is that comorbid mental disorders can be more adequately targeted because these protocols usually focus on what these disorders have in common, rather than on disorder-specific symptom variations [17, 22, 26, 28]. For instance, extensive research shows the key role played by neuroticism in the onset and maintenance of both anxiety and depressive disorders, indicating its relevance in research and clinical practice [15–17, 29, 30]. In this regard, the “shared mechanisms approach”, described by Sauer-Zavala et al. [13], is based on the assumption that there are core mechanisms underlying both anxiety and depressive disorders, and that, consequently, in order for the specific symptoms to improve (e.g. symptoms of panic, symptoms of social anxiety, and so on), treatment should focus on addressing these common processes. Based on this approach, some authors have argued that a transdiagnostic treatment may be appropriate for a wide range of disorders, including all the anxiety and unipolar mood disorders, and even somatoform and dissociative disorders [15, 22], while facilitating the treatment of patients with comorbidity [12]. There are, nevertheless, other transdiagnostic approaches to the treatment of ED (including the treatment of comorbid presentations), such as individually-tailored CBT [20] or “third wave” therapies (e.g. mindfulness and acceptance and commitment therapy) [31–33]. Finally, transdiagnostic treatments also have the potential to address “not otherwise specified” (NOS) diagnoses for which there are no evidence-based treatments in the literature (e.g. anxiety NOS) [13].

There is a growing body of literature on the efficacy and effectiveness of transdiagnostic treatments for ED. To date, various meta-analyses have shown the efficacy of these treatments in adults with ED, compared to control conditions, on measures of overall anxiety [34–38] and disorder-specific anxiety [38], as well as depression [35–38] and quality of life [36–38]. Moreover, a meta-analysis of the efficacy of these protocols, compared to disorder-specific CBT, found no significant differences in the efficacy of these two treatment approaches on anxiety outcomes [39]. Nevertheless, no prior study has examined how comorbidity is reported and assessed in trials analyzing transdiagnostic protocols, despite the importance of comorbidity in aspects such as the clinical severity, the clinical course, and the rate of relapse in patients with comorbid anxiety and depressive disorders [8, 10, 11]. Some studies on transdiagnostic treatments for ED have assessed treatment effects on comorbid symptoms, as well as the symptoms primarily targeted in the study. For instance, some studies include self-reported measures to assess a range of comorbid disorder-specific symptoms [21, 40, 41], and others assess the impact of the intervention in terms of the number of comorbid disorders, in addition to the number of principal diagnoses [22]. However, this aspect has not yet been systematically analyzed in the literature on transdiagnostic treatments for ED.

Regarding the types of diagnoses targeted by transdiagnostic treatments, the transdiagnostic treatments published to date may range from those targeting only two disorders [42–44] to those addressing a larger number of ED [45–47]. Moreover, transdiagnostic treatments may focus on anxiety disorders alone [48–50], or anxiety disorders along with depressive disorders [51–53]. There is, therefore, great disparity in the types and frequencies of anxiety and depressive disorders targeted in transdiagnostic treatments. However, to our knowledge, the real distribution of specific diagnoses in these interventions, i.e. the classes of disorders and the most frequent and infrequent disorders targeted in transdiagnostic treatments for ED, has not yet been analyzed.

Taking all this into consideration, a systematic review was conducted to answer the following research questions: a) Are comorbid disorders evaluated in transdiagnostic treatments for emotional disorders? b) What diagnoses are targeted in transdiagnostic treatments for emotional disorders? and c) What is the real distribution of the diagnoses at baseline in transdiagnostic treatments for emotional disorders?

## Methods

### Search strategy, data extraction, and coding

A systematic search of the peer-reviewed literature was conducted through the following databases: PsycINFO, PubMed, EMBASE and the Cochrane Register of Controlled Trials. The following terms were combined to conduct the search: “transdiagnostic”, “unified”, “mixed anxiety and depression”, “mixed depression and anxiety”, “heterogeneous” “depression”, “anxiety”. The deadline for inclusion of studies was February 6^th^ (2018) (with no limits applied for year of publication). The systematic review protocol was registered in the International Prospective Register of Systematic Reviews (PROSPERO, CRD42018088138). Studies were included based on the following eligibility criteria:

- The study was a randomized controlled trial (RCT) that was compared to one of the following conditions: a waiting list control condition, placebo, attention control condition, active control condition (i.e. other treatment), and care as usual/treatment as usual control condition.
- The study was written in English.
- Participants were adults (18 years old and older).
- Participants had at least a principal diagnosis of an anxiety disorder or a score above a cutoff point on an anxiety self-report scale, and/or a principal diagnosis of a depressive disorder or a score above a cutoff point on a depression self-report scale.
- The study evaluated a transdiagnostic treatment for anxiety disorders and/or depression (i.e. unipolar mood disorders, anxiety disorders, posttraumatic stress disorder, and obsessive-compulsive disorder). To be included in the systematic review, the intervention had to target at least two different anxiety disorders or an anxiety disorder in addition to a depressive disorder.

Two assessors (AG-R and AD-G) conducted the review and selection of studies independently. The final selection of the included studies was supervised by a third expert evaluator (CB).

The following variables were included: a) study (authors and year of publication); b) country; c) aims of the study; d) hypotheses (when available); e) setting (e.g. community, primary care) and delivery format (e.g. Internet, face-to-face, individual, group); f) inclusion criteria regarding the types of diagnoses or symptoms targeted (“or” when the participants had to have at least one of the disorders, and “+” when the participants had to have both disorders); g) groups (sample size); percentage of females; and i) the distribution of each type of diagnosis at baseline. In order to evaluate the data on comorbidity, three dichotomous variables (yes/no) were created and added to the table: a) whether a principal diagnostic or symptom complaint was reported (e.g. main complaint of generalized anxiety symptoms); b) whether comorbid disorders and/or symptoms were reported. To belong to this category, the study had to report at least the proportion of patients presenting comorbid disorders or symptoms (e.g. the number of patients with one comorbid disorder, two comorbid disorders, and so on); and c) whether treatment response on comorbid disorders/symptoms was evaluated, i.e. a diagnosis made using a diagnostic interview or the severity of the disorder or symptoms through scales. All the aforementioned variables were extracted and coded independently by AG-R and AD-G, and disagreements were solved by discussion with a third author (CB).

### Definition of emotional disorders included in the study

ED were considered for this study following the criteria of the Diagnostic and Statistical Manual of mental disorders, 4^th^ edition (DSM-IV-TR) [54] and the definitions of these disorders adopted by previous authors [15], namely, unipolar mood disorders and anxiety disorders. Unipolar mood disorders included major depressive disorder (MDD), dysthymic disorder (D), and depression not otherwise specified (Depression NOS), whereas anxiety disorders included generalized anxiety disorder (GAD), panic disorder with or without agoraphobia (PD/AG), social anxiety disorder (SAD), posttraumatic stress disorder (PTSD), obsessive-compulsive disorder (OCD), specific phobia (SP), and anxiety disorder not otherwise specified (Anxiety NOS). Although the classification of some of these disorders has changed with the publication of the DSM-5 [55] (i.e. PTSD and OCD are no longer considered anxiety disorders), the DSM-IV-TR was followed because most of the studies analyzed had recruited participants based on this diagnostic manual.

### Quality assessment

The quality of the included studies was assessed using four items from the Cochrane Collaboration Risk of bias tool [56], which estimates potential bias in randomized controlled trials, including the following domains: random sequence generation, allocation concealment, blinding of outcome assessment (if applicable), and handling of incomplete outcome data. Each item on the tool was rated as low, high, or, in the case of insufficient information, unclear risk. This process was conducted by two independent researchers (AG-R and AD-G). Disagreements were resolved through discussion and, when necessary, by asking a senior researcher (CB).

## Results

### Selection and inclusion of studies

**Fig 1:**
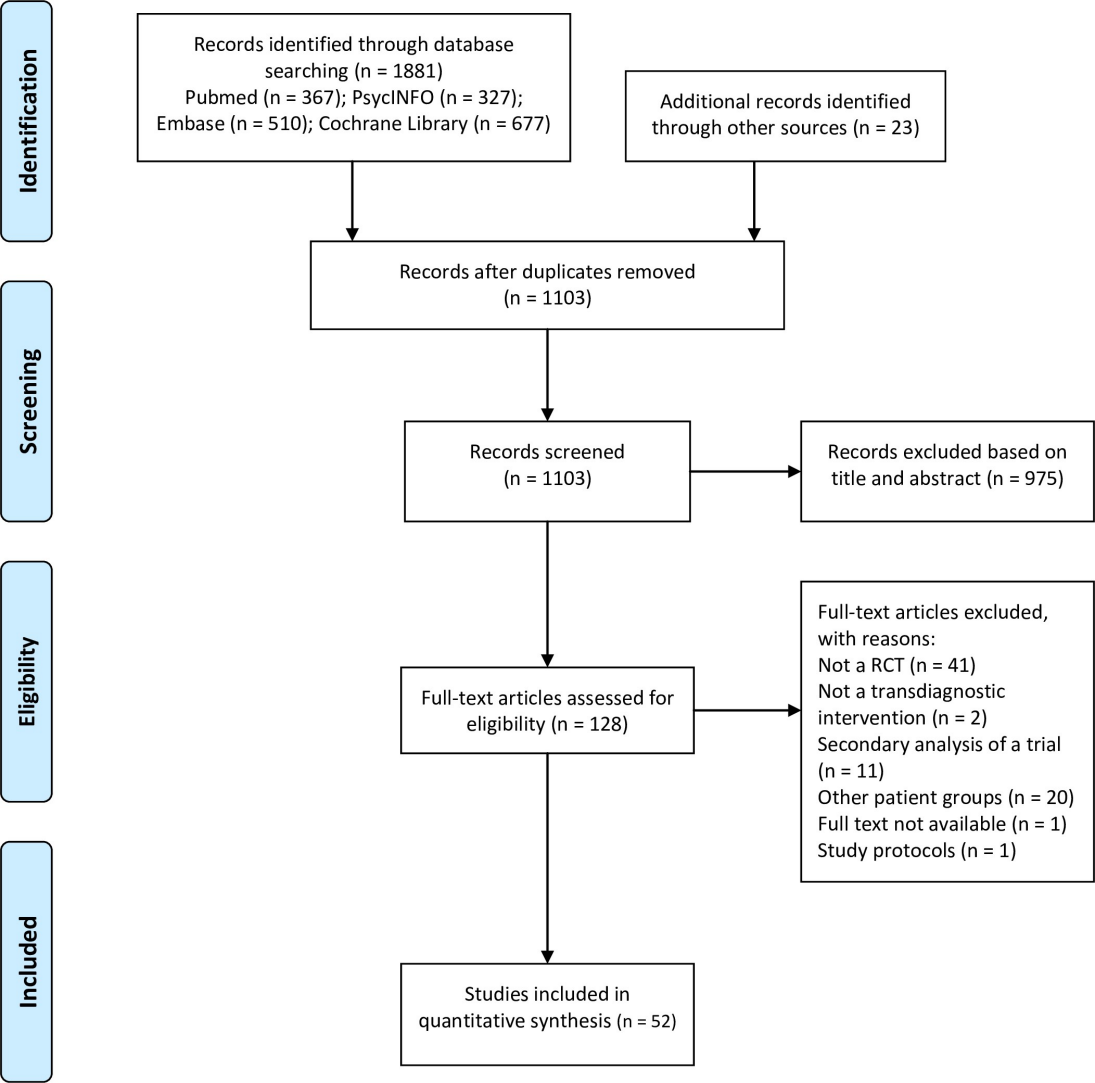
PRISMA flowchart.

The study selection process is presented in the PRISMA flowchart (Fig 1). A total of 1881 studies were identified through database searches (Pubmed = 367; PsycINFO = 327; Embase = 510; Cochrane Library = 677), and 23 additional records were identified through other sources (i.e. meta-analyses about the efficacy of transdiagnostic treatments for anxiety and depression). After removing duplicates, 1103 records were screened based on title and abstract. Of them, 128 full-articles were assessed for eligibility, of which 52 were selected for final inclusion in the systematic review.

### Characteristics of included studies

Relevant characteristics of the included studies are shown in Table 1. All the studies were randomized controlled trials with a total of 7007 participants. Most of the studies were conducted in the United States (n = 19, 37%), Australia (n = 12, 23%), Sweden (n = 6, 12%), and the United Kingdom (n = 5, 10%). The most common setting was the community (37 studies, 71%), followed by primary care (6 studies, 12%), specialized care (4 studies, 8%), community/primary care (3 studies, 6%) [57–59], and university students (2 studies, 4%) [60, 61]. Regarding the delivery format (i.e. face-to-face vs. web-based/computerized; individual vs. group), 24 treatments were delivered face-to-face (46%), 23 were Internet-based (44%), 4 were computerized (8%) [25, 62–64], and 1 was delivered by telephone (2%) [58]. Of the 52 studies, 38 were delivered in an individual format (73%), whereas 13 were delivered in a group format (25%), and one combined individual and group formats (2%) [65]. Regarding the control conditions, 21 studies used an active control condition (of which 8 were disorder-specific treatments), 19 studies used a waiting list control, 8 employed a care as usual/treatment as usual condition, 4 used an attention control condition [20, 64, 66, 67], and 1 employed a placebo control condition [50]. Finally, only 1 cost-effectiveness study was identified [67].

**Table 1:** Characteristics of the included studies.

Study	Ctry	Aims	Hypotheses	Setting & Delivery format	Targeted diagnoses (inclusion criteria)	Groups (n)	Age	% female	Diagnoses (distribution) at baseline	Principal diagnosis reported	Comorb reported	Comorb assessed
Arch et al., 2012 [31]	US	To compare ACT and CBT in a sample with multiple anxiety disorders.	ACT would improve cognitive flexibility and valued living to a greater degree than CBT.	C F2F Indv	D/AG, SAD, SP, OCD, or GAD	1. ACT (57) 2. CBT (71)	37,93 (11,70)	52,3	PD/AG (N = 53) SAD (N = 25) GAD (N = 26) OCD (N = 17) SP (N = 6)	Yes	Yes	Yes
Arch et al., 2013 [32]	US	To compare MBSR and CBT in the treatment of anxiety disorders.	1. CBT would improve anxiety symptoms to a greater degree than MBSR 2. MBSR would improve broader symptoms (depression and co-occurring emotional disorders) to a greater degree than CBT.	SP F2F Group	PD/AG, SAD, SP, OCD, GAD, or PTSD	1. MBSR (45) 2. CBT (60)	45,91 (13,68)	17	PD/AG (N = 31) GAD (N = 38) SAD (N = 16) PTSD (N = 15) OCD (N = 5)	Yes	Yes	Yes
Barlow et al., 2017 [18]	US	To explore whether the UP is at least as efficacious as single-disorder protocols in the treatment of anxiety disorders.	The UP would be at least as efficacious as single-disorder CBT at post-treatment and at 6-month follow-up.	C F2F Indv	PD/AG, SAD, OCD, or GAD	1. UP (88) 2. SD-CBT (91) 3. WLC (44)	31,10 (11,0)	55,6	OCD (N = 44) GAD (N = 62) PD/AG (N = 59) SAD (N = 58)	Yes	Yes	Yes
Barrowclough et al., 2001 [57]	UK	To compare CBT and SC in older adults with anxiety disorders.	N/A	PC + C F2F Indv	PD/AG, SAD, GAD, or Anx NOS	1. CBT (19) 2. SC (24)	72 (6,2)	77	PD/AG (N = 22) SAD (N = 1) GAD (N = 8) Anx NOS (N = 12)	Yes	Yes	No
Berger et al., 2014 [19]	CH	To compare T-CBT for symptoms of SAD, PD/AG, and GAD to SD-CBT and a WLC.	1. To study whether T-CBT outpferforms SD-CBT. 2. To analyze whether both active treatment conditions outperform the WLC.	C Internet Indv	SAD, PD/AG, or GAD	1. T-CBT (44) 2. SD-CBT (44) 3. WLC (44)	35,1 (11,14)	56	SAD (N = 113) PD/AG (N = 44) GAD (N = 33)	No	Yes	Yes
Berger et al., 2017 [48]	CH	To compare CBT+CAU for anxiety disorders to CAU in PC.	CBT + CAU would reduce anxiety and related symptoms to a greater degree than CAU in patients with SAD, PD/AG and/or GAD.	PC Internet Indv	SAD, PD/AG, or GAD	1. CBT + CAU (70) 2. CAU (69)	42 (12,1)	70,5	SAD (N = 40) PD/AG (N = 63) GAD (N = 36)	Yes	Yes	Yes
Boettcher et al., 2014 [68]	DE	To compare MT to an online DF for SAD, PD, GAD, and/or Anx NOS.	MT would improve anxiety, depression, insomnia, and quality of life to a greater degree than the online DF.	C Internet Indv	SAD, PD/AG, GAD, or Anx NOS	1. MT (45) 2. Online DF (46)	38 (10,3)	71,4	GAD (N = 17) SAD (N = 26) PD (N = 30) Anx NOS (N = 18)	Yes	Yes	No
Bolton et al., 2014^a^ [42]	US	To test transdiagnostic CBT for comorbid presentations of depression, anxiety, and trauma symptoms among trauma survivors in a low-resource setting.	N/A	C F2F Indv	Dep or PTSD	1. CBT (182) 2. WLC (165)	1: 36,5 (12,6) 2: 34,3 (11,4)	63	PTSD/Dep (N = 347)	No	No	No
Brenes et al., 2012 [58]	US	To compare CBT-T and IO for the treatment anxiety disorders in older adults.	CBT-T would improve anxiety, worry, depressive symptoms, and quality of life to a greater degree than IO.	PC + C T Indv	GAD, PD, or Anx NOS	1. CBT-T (30) 2. IO (30)	1: 68,8 (7,3) 2: 69.5 (6.9)	83,3	GAD (N = 30) GAD+PD (N = 25) PD (N = 3) Anx NOS (N = 2)	Yes	Yes	Yes
Bressi et al., 2010 [69]	IT	To compare STPP and TAU in the treatment of patients with anxiety or depressive disorders.	1. STPP would produce equal or greater reductions in psychiatric symptoms than TAU. 2. Patients in STPP would show fewer interpersonal problems than patients in TAU at post-treatment.	SC F2F Indv	GAD, PD, SAD, MDD, or DD	1. STPP (30) 2. TAU (30)	1: 35,75 (9,25) 2: 38,67 (9,28)	76,7	Dep (N = 21) PD (N = 15) SAD (N = 8) GAD (N = 16)	Yes	No	No
Carlbring et al., 2011 [20]	SE	To compare T-CBT to an attention control condition (online discussion group) in anxiety disorders.	T-CBT would reduce symptoms of anxiety and mood, and increase quality of life.	C Internet Indv	Any specific anxiety disorder, or Anx NOS	1. T-CBT (27) 2. AC (27)	38,8 (10,7)	76	Dep (N = 23) PD (N = 5) PD+AG (N = 12) OCD (N = 1) PTSD (N = 1) SAD (N = 21) GAD (N = 11) Anx NOS (N = 7)	No	Yes	No
Craske et al., 2007 [65]	US	To compare T-CBT for the treatment of principal PD/AG + CBT for the comorbid condition to CBT focused only on PD/AG	1. CBT would improve symptoms of PD/AG to a greater degree than T-CBT 2. CBT would improve comorbid symptoms to a greater degree than T-CBT	C F2F Indv /group	PD/AG + 1 anxiety disorder/mood disorder	1. CBT (33) 2. T-CBT (32)	36,8 (9,1)	60	PD/AG (N = 65)	Yes	Yes	Yes
Day et al., 2013^b^ [60]	CA	To compare CBT for the treatment of anxiety, depression and/or stress to a WLC in university students.	1. CBT would improve anxiety, depression and stress symptoms to a greater degree than the WLC. 2. The improvements would be maintained at a 6-month follow-up.	Univ stud Internet Indv	Symptoms of depression, anxiety or stress	1. CBT (33) 2. WLC (33)	23,55 (4,98)	89,3	Participants had symptoms of anxiety, stress, and/or depression (information on diagnoses unavailable)	No	No	No
Dear et al., 2015 [21]	AU	To compare transdiagnostic CBT for GAD and comorbid symptoms to SD-CBT, in terms of relative efficacy and acceptability when provided in both clinician-guided and self-guided formats.	1. Transdiagnostic CBT and SD-CBT would improve symptoms of GAD. 2. TD-CBT would improve symptoms of comorbid Dep, SAD and PD at each time point to a greater degree than SD-CBT.	C Internet Indv	Symptoms of GAD	1. CBT (170) 2. SD-CBT (168)	43,78 (11,29)	76	GAD (N = 338)	Yes	Yes	Yes
Dear et al., 2016 [35]	AU	To compare transdiagnostic CBT for SAD and comorbid symptoms to SD-CBT, in terms of efficacy and acceptability when provided in both clinician-guided and self-guided formats.	1. Transdiagnostic CBT and SD-CBT would improve symptoms of SAD similarly. 2. Transdiagnostic CBT would reduce symptoms of comorbid Dep, GAD and PD at each time point to a greater degree than SD-CBT.	C Internet Indv	Symptoms of SAD	1. CBT (105) 2. SD-CBT (115)	41,57 (10,89)	58	SAD (N = 220)	Yes	Yes	Yes
Ejeby et al., 2014 [70]	SE	To compare CBT and MMI to CAU alone for patients with anxiety, depressive, and stress-related disorders.	CBT and MMI would improve quality of life and psychological symptoms to a greater degree than CAU alone.	PC F2F Group	Depression, anxiety, stress, or somatoform disorders	1. CBT + CAU (84) 2. MMI + CAU (80) 3. CAU (81)	1: 43,3 (10,3) 2: 44,3 (9,5) 3: 45,0 (9,5)	80,8	Dep (N = 139) Anx disorders (N = 81) Somatoform disorders (N = 10) Eating disorders (N = 6) AUD (N = 2)	No	No	No
Erickson et al., 2007 [71]	CA	To compare CBT for different anxiety disorders to a WLC.	1. CBT would improve anxiety symptoms to a greater degree than the WLC. 2. CBT would improve within-group symptoms of anxiety at post-treatment and follow-up.	C F2F Group	PD/AG, OCD, SAD, GAD, SP, or PTSD	1. CBT (73) 2. WLC (79)	1: 40,7 (11,8) 2: 41,0 (11,1)	63,8	SAD (N = 46) PD/AG (N = 36) GAD (N = 31) PSTD (N = 16) OCD (N = 16) SP (N = 7)	Yes	Yes	No
Farchione et al., 2012 [22]	US	To compare the UP for anxiety disorder to a WLC.	1. The UP would be efficacious in improving the symptoms of patients with GAD, SAD, PD/AG, and OCD. 2. The UP would reduce the severity of comorbid disorders at both post-treatment and follow-ups.	C F2F Indv	Anxiety disorders	1. CBT (26) 2. WLC (11)	1: 29,38 (9,86) 2: 30,64 (9,15)	59,5	GAD (N = 7) SAD (N = 8) OCD (N = 8) Anx NOS (N = 2) PDA (N = 8) PTSD (N = 1) SAD+Anx NOS (N = 1) GAD+SAD (N = 1) OCD+PD/AG (N = 1)	Yes	Yes	Yes
Fogliati et al., 2016 [23]	AU	To compare transdiagnostic CBT for PD and comorbid symptoms to SD-CBT in terms of efficacy and acceptability when provided in both clinician-guided and self-guided formats.	1. Transdiagnostic CBT and SD-CBT would improve symptoms of PD similarly. 2. Transdiagnostic CBT would reduce symptoms of comorbid Dep, GAD, and SAD at each time point to a greater degree than SD-CBT.	C Internet Indv	Symptoms of PD	1. CBT (72) 2. SD-CBT (73)	41,40 (11,28)	79	PD (N = 145)	Yes	Yes	Yes
Forman et al., 2007 [51]	US	To compare ACT and CBT in the treatment of anxiety and depression.	1. CBT would show stronger mediation effects for the ability to identify and report on internal experiences than ACT. 2. ACT would show stronger mediation effects for experiential acceptance and current-moment awareness than CBT.	C F2F Indv	Symptoms of anxiety and/or depression	1. ACT (55) 2. CBT (44)	27,87 (7,25)	80,2	Dep (N = 34) Anxiety disorder (N = 32) AD (N = 10)	No	No	No
Hadjistavro- poulos et al., 2017 [72]	CA	To compare CBT + standard support to CBT + optional support in the treatment of anxiety and depression.	1. CBT + optional support would not be inferior to CBT + standard support. 2. CBT + optional support and CBT+ standard support would be similar in terms of symptom improvement, completion rates, and satisfaction with the treatment.	C Internet Indv	Symptoms of anxiety and/or depression	1. CBT + standard support (92) 2. CBT + optional support (88)	38,29 (12,92)	78,7	Dep (N = 97) GAD (N = 100) PD (N = 80) SAD (N = 96)	No	Yes	Yes
Johansson et al., 2012 [73]	SE	To compare T-CBT for anxiety and comorbid symptoms to CBT, and to an active control group (Online DF focused on depression).	1. T-CBT and CBT would produce improvements. 2. T-CBT would produce greater improvements than CBT. 3. An effect was expected on the online DF, but smaller than in the CBT treatment groups.	C Internet Indv	MDD	1. T-CBT (39) 2. CBT (40) 3. Online DF (42)	44,7 (12,1)	71,1	Dep (N = 121)	Yes	Yes	No
Johansson et al., 2013 [46]	SE	To compare PP and SC in patients with depression and anxiety disorders.	1. PP would improve measures of depression and anxiety to a greater degree than SC. 2. Larger effects were expected on measures of depression in patients with depression as their principal diagnosis compared to patients who did not have depression as their principal diagnosis. 3. Larger effects were expected on measures of anxiety in patients with anxiety as their principal diagnosis compared to patients who did not have an anxiety disorder as their principal diagnosis.	C Internet Indv	MDD, SAD, PD, GAD, Anx NOS, or Dep NOS	1. PP (50) 2. SC (50)	44,9 (13,1)	82	Dep (N = 72) GAD (N = 49) SAD (N = 36) PD (N = 19) Anx/Dep NOS (N = 4)	No	Yes	Yes
Johnston et al., 2011 [50]	AU	To compare Clinician-guided CBT and Coach-guided CBT to a WLC.	1. The pooled Clinician-guided and Coach-guided groups would improve in general and on disorder-specific measures of anxiety, depression, and disability to a greater degree than the WLC. 2. Participants in the CBT groups would rate the treatment as acceptable. 3. The pooled Clinician-guided and Coach-guided groups would show significant improvement on disorder-specific measures of anxiety over time. 4. Participants in both CBT groups would show similar outcomes on all measures and at all measurement points.	C Internet Indv	GAD, SAD, or PD/AG	1. Clinician guided CBT (46) 2. Coach guided CBT (43) 3. WLC (42)	41,62 (12,83)	58,8	GAD (N = 59) SAD (N = 45) PD/AG (N = 27)	Yes	Yes	Yes
Kim et al., 2009 [43]	KR	To compare MBCT to a Psychoeducation control group in patients with PD and GAD.	N/A	SC F2F Group	GAD, or PD/AG	1. MBCT (24) 2. Psychoeduc (22)	1: 40,8 (7,3) 2: 38,1 (9,7)	37	GAD (N = 11) PD (N = 35)	Yes	No	No
Lang et al., 2017 [59]	US	To compare ACT and P-CT in veterans with anxiety or depressive disorders, or those with postconcussive symptoms.	N/A	PC + C F2F Indv	Anxiety or depressive disorder	1. ACT (80) 2. P-CT (80)	34,2 (8)	20	Dep (N = 97) PTSD (N = 131) PD/AG (N = 124) SAD (N = 26) OCD (N = 21) GAD (N = 32) Anx NOS (N = 6)	No	No	No
Marks et al., 2004 [62]	UK	To compare Comp SE and face-to-face SE to a placebo group (relaxation) in patients with phobias or panic disorder.	1. Comp-SE would show similar efficacy to face-to-face SE. 2. Both SE groups would be more effective than Comp Self-Relaxation.	SC Comp Indv	PD/AG, SAD, or SP	1. Comp SE (37) 2. Face-to-face SE (39) 3. Comp Self- Relaxation (17)	38 (12)	69	PD+AG (N = 24) AG (N = 3) SAD (N = 24) SP (N = 39)	Yes	Yes	No
Mullin et al., 2015 [61]	AU	To compare CBT for university students with stress, anxiety, low mood, and depression to WLC, in terms of both efficacy and acceptability.	1. CBT would reduce symptoms of anxiety and depression at post-treatment to a greater degree than the WLC. 2. Participants with clinical levels symptoms would show improvements consistent with those found in prior studies on Internet CBT. 3. Symptom improvements would be maintained at 3-month follow-up. 4. Participants would be satisfied with the treatment.	Univ stud Internet Indv	Symptoms of anxiety or depression	1. CBT (30) 2. WLC (23)	1: 28,6 (10,05) 2: 26,9 (11,51)	64,2	GAD (N = 40) PD (N = 12) SAD (N = 19) Dep (N = 18)	No	Yes	No
Neacsiu et al., 2014 [66]	US	To compare DBT-ST for emotion dysregulation to an activities-based support group in order to: 1. Explore the effects of DBT-ST on anxiety and depression. 2. Investigate the mediation effects of DBT skills use on differential changes. 3. Explore whether confounding effects accounted for any significant outcomes. 4. Explore the feasibility of DBT-ST in terms of retention rates, treatment credibility and satisfaction, and compliance with the treatment protocol.	1. DBT-ST would reduce emotion dysregulation to a greater degree than the activities-based support group. 2. The use of DBT skills would mediate the differential changes between groups.	C F2F Group	Anxiety or depressive disorder	1. DBT (22) 2. AC (22)	1: 32,37 (10,50) 2: 38,82 (13,55)	65,9	Dep (N = 34) PD (N = 6) AG (N = 3) GAD (N = 29) SAD (N = 16) SP (N = 8) OCD (N = 5) PTSD (N = 4) Anx NOS (N = 4) SUD (N = 3)	No	Yes	No
Newby et al., 2013 [52]	AU	To compare CBT for mixed GAD and MDD to a WLC.	CBT would show greater improvements than the WLC.	C Internet Indv	Symptoms of anxiety + depression	1. CBT (46) 2. WLC (53)	44,3 (12,2)	77,8	GAD/MDD (N = 47) GAD (N = 37) MDD (N = 15)	Yes	Yes	Yes
Nordgren et al., 2014 [67]	SE	To compare CBT to an AC group in terms of cost-effectiveness on anxiety disorders.	1. CBT would be moderately more effective than the AC group both at post-treatment and at 1-year follow-up. 2. CBT would be cost-effective.	PC Internet Indv	Anxiety disorders	1. CBT (50) 2. AC (50)	1: 35 (13) 2: 36 (12)	63	GAD (N = 10) SAD (N = 32) PD/AG (N = 31) AG (N = 8) Anx NOS (N = 19)	Yes	Yes	No
Norton, 2012 [28]	US	1. To compare CBT to relaxation in terms of overall efficacy. 2. To compare CBT to relaxation on treatment credibility and acceptability. 3. To compare CBT effects across diagnoses to analyze differential efficacy by diagnosis.	1. Participants in both groups would show significant improvements in anxiety over the course of treatment. 2. CBT would show equivalence/non inferiority with relaxation. 3. Participants would not show differences in outcomes by primary or secondary diagnosis.	C F2F Group	Anxiety disorders	1. CBT (65) 2. Relaxation (22)	32,98 (10,73)	62,1	SAD (N = 37) PD/AG (N = 31) GAD (N = 15) Anx NOS (N = 2) OCD (N = 1) SP (N = 1)	Yes	Yes	Yes
Norton & Hope, 2005 [24]	US	1. To compare CBT to a WLC in patients with different anxiety disorders.	1. CBT would produce significant improvements on diagnostic indices. 2. CBT would show significant reductions at post-treatment on measures of anxiety, whereas no improvement would be observed in the WLC on these measures. 3. CBT would improve measures of the common core psychopathology during the second phase of treatment, whereas no improvement would be observed in the WLC on these measures.	C F2F Group	Anxiety disorders	1. CBT (12) 2. WLC (12)	39,58 (11,88)	60,9	SAD (N = 5) PD/AG (N = 4) GAD (N = 10) OCD (N = 3) PD (N = 1) PTSD (N = 1)	Yes	Yes	No
Norton & Barrera, 2012 [49]	US	To compare transdiangostic CBT to SD-CBT for PD, GAD, and SAD.	Both conditions would significantly improve anxiety over the course of treatment, and these results in both conditions would be non-inferior.	C F2F Group	PD, SAD, or GAD	1. CBT (23) 2. SD-CBT (23)	31,46 (8,93)	50	SAD (N = 25) GAD (N = 10) PD (N = 11)	Yes	Yes	No
Proudfoot et al., 2003 [25]	UK	To compare CBT to TAU in patients with anxiety, depression, or mixed anxiety and depression.	CBT would produce greater improvements than TAU.	PC Comp Indv	Depression, mixed anxiety-depression, or anxiety disorders	1. CBT (88) 2. TAU (77)	1: 43,7 (14,7) 2: 45,7 (14,1)	73,7	Mixed anx-dep (N = 80) Dep (N = 61) PD (N = 10) SP (N = 4) AG (N = 5) SP (N = 5)	Yes	No	No
Proudfoot et al., 2004 [63]	UK	1. To compare CBT to TAU in patients with anxiety, depression, or mixed anxiety and depression in terms of efficacy. 2. To investigate interactions of CBT with clinical, demographic, and setting variables.	N/A	PC Comp Indv	Depression, mixed anxiety-depression, or anxiety disorders	1. CBT (145) 2. TAU (128)	1: 43,6 (14,3) 2: 43,4 (13,7)	73,7	Mixed anx-dep (N = 142) Dep (N = 92) PD (N = 14) SP (N = 11) AG (N = 8) SP (N = 6)	Yes	No	No
Riccardi et al., 2017 [74]	US	To compare FSBET to a WLC.	1. FSBET would improve overall outcome to a greater degree than the WLC. 2. FSBET would produce clinically significant improvements on principal diagnosis and secondary diagnosis symptoms. 3. Improvements in the FSBET group would be maintained at 1-month follow-up. 4. The relationship between pre- and post-treatment changes would be mediated by the reduction in safety aid use.	C F2F Indv	PD/AG, SAD, or GAD	1. FSBET (16) 2. WLC (12)	28,6 (11,8)	75	GAD (N = 9) PD (N = 8) SAD (N = 11)	Yes	Yes	Yes
Roy-Byrne et al., 2010 [47]	US	To compare CBT to CAU in patients with PD, GAD, SAD, or PTSD.	CBT would reduce symptoms of anxiety, and improve measures of health-related quality of life, functioning, and quality of care delivered to a greater degree than CAU.	PC Internet Indv	PD, GAD, SAD, or PTSD	1. CBT (503) 2. CAU (501)	43,47 (13,4)	71,1	PD (N = 475) GAD (N = 756) SAD (N = 405) PTSD (N = 181) Dep (N = 648)	No	Yes	No
Schmidt et al., 2012 [75]	US	To compare FSBET to a WLC in patients with multiple anxiety disorders.	1. FSBET would improve in overall outcomes to a greater degree than the WLC. 2. FSBET would show efficacy on each of the anxiety disorders evaluated. 3. Improvements in the FSBET group would be maintained at 6-month follow-up.	C F2F Group	PD/AG, SAD, or GAD	1. FSBET (57) 2. WLC (39)	36,3 (10,7)	72	GAD (N = 26) PD (N = 36) SAD (N = 34)	Yes	Yes	No
Schmidt et al., 2017 [64]	US	To compare CAST + CBM to PHET + sham CBM in patients with co-ocurring anxiety and suicidal ideation.	1. CAST + CBM would improve overall anxiety sensitivy and the cognitive dimension of anxiety sensitivity to a greater degree than PHET + sham CBM. 2. Reductions in anxiety sensitivity would be maintained at the 4-month follow-up. 3. Changes in anxiety sensitivity would affect symptoms of suicidal ideation at the follow-up period.	C Comp Indv	Clinical anxiety sensitivity + Suicidal ideation + Anxiety or depressive disorder	1. CAST+CBM (37) 2. PHET+sham CBM (AC) (37)	30,77 (14,16)	75,6	PD (N = 7) SAD (N = 10) OCD (N = 1) PTSD (N = 11) GAD (N = 2) Anx/Dep NOS (N = 2) Dep (N = 37)	Yes	No	No
Schneider et al., 2005 [76]	UK	To compare CBT to minimal CBT in the treatment of PD/AG, SAD, and SP.	CBT would improve phobia/panic to a greater degree than minimal CBT at post-treatment and follow-up.	C Internet Indv	PD/AG, SAD, or SP	1. CBT (45) 2. Minimal CBT (23)	39 (11)	74	PD+AG (N = 25) AG (N = 2) SAD (N = 24) SP (N = 17)	Yes	Yes	Yes
Schröder et al., 2017 [77]	DE	1. To compare CBT to CAU in individuals with panic and phobias. 2. To explore differences in treatment effects by diagnosis. 3. To explore treatment moderators.	N/A	C Internet Indv	PD/AG, SAD, or SP	1. CBT (89) 2. CAU (90)	1: 36,5 (9,95) 2: 36,5 (10,26)	72	PD (N = 91) AG (N = 119) PD+AG (N = 73) SAD (N = 98) SP (N = 66)	No	No	No
Silfvernagel et al., 2012 [78]	SE	To compare T-CBT to a WLC in patients with panic symptoms with comorbid anxiety and depressive symptoms, in two age groups (18–30 and 31–45 years old).	1. T-CBT would produce decreases in measures of panic, anxiety, and depression. 2. T-CBT would increase quality of life. 3. The effects of T-CBT would be maintained at 12-month follow-up. 4. No significant differences would be observed between the two age groups.	C Internet Indv	Recurrent panic attacks	1. T-CBT (29) 2. WLC (28)	32,4 (6,9)	65	PD (N = 4) PD+AG (N = 47) GAD (N = 11) SAD (N = 9) Anx NOS (N = 1) Dep (N = 5)	No	Yes	No
Taylor et al., 2017 [79]	US	To compare PAI to a WLC in individuals with anxiety or depression.	N/A	C F2F Indv	Anxiety or depressive symptoms	1. PAI (16) 2. WLC (13)	1: 29,8 (12,2) 2: 29,0 (12,0)	60,7	MDD (N = 16) SAD (N = 16) GAD (N = 11) PTSD (N = 6) PD (N = 2) OCD (N = 1) Eating disorder (N = 3) AUD (N = 2) SUD (N = 1)	No	Yes	No
Titov et al., 2010 [26]	AU	1. To compare CBT to a WLC in individuals with PD/AG, GAD, and/or SAD. 2. To analyze whether additional gains would be shown by the WLC after mofifying the treatment program with the feedback of the patients in the treatment group.	1. CBT would improve measures of overall and disorder-specific anxiety, depression, neuroticism, and disability to a greater degree than the WLC. 2. Participants allocated to CBT would rate the procedure as acceptable.	C Internet Indv	GAD, SAD, or PD	1. CBT (40) 2. WLC (38)	39,5 (13,0)	67,9	GAD (N = 34) PD/AG (N = 21) SAD (N = 23)	Yes	Yes	Yes
Titov et al., 2011 [27]	AU	To compare CBT to a WLC in patients with GAD, SAD, and/or PD/AG.	1. CBT would improve generic measures of depression and anxiety, neuroticism, and disability to a greater degree than the WLC 2. Fewer patients would meet the diagnostic criteria for MDD, GAD, SAD, or PD/AG in the treatment group 3. Participants allocated to CBT would rate the procedure as acceptable.	C Internet Indv	Depression, GAD, SAD, or PD/AG	1. CBT (37) 2. WLC (37)	43,9 (14,6)	73	Dep (N = 38) GAD (N = 21) PD/AG (N = 7) SAD (N = 8)	Yes	Yes	Yes
Titov et al., 2013 [80]	AU	1. To compare CBT + automated emails to CBT alone for symptoms of anxiety and depression in terms of clinical outcomes and adherence. 2. To provide preliminary data on safety and acceptability.	1. CBT + automated emails would produce better completion rates and reductions in clinical outcomes than CBT alone. 2. CBT + automated emails would be more beneficial for more severe patients.	C Internet Indv	Depression, GAD, SAD, or PD	1. CBT+ autom emails (100) 2. CBT only (106) 3. WLC (51)	41,30 (9,76)	73,5	Dep (N = 85) GAD (N = 84) PD (N = 34) SAD (N = 54)	Yes	No	No
Titov et al., 2015 [41]	AU	To compare transdiagnostic CBT for depression and comorbid symptoms toSD-CBT in terms of efficacy and acceptability when provided in both clinician-guided and self-guided formats.	1. Transdiagnostic CBT and SD-CBT would improve symptoms of depression similarly. 2. Transdiagnostic CBT would reduce symptoms of comorbid PD, GAD, and SAD at each time point to a greater degree than SD-CBT.	C Internet Indv	Depression symptoms	1. CBT (149) 2. SD-CBT (141)	44,19 (11,75)	72	Dep (N = 290)	Yes	Yes	Yes
Vøllestad et al., 2011 [45]	NO	To compare MBSR to a WLC in patients with PD/AG, SAD, and GAD.	N/A	C F2F Group	PD/AG, SAD,or GAD	1. MBSR (39) 2. WLC (37)	42,5 (11,3)	67,1	PD/AG (N = 38) SAD (N = 25) GAD (N = 13)	Yes	Yes	No
Wetherell et al., 2009 [44]	US	To compare MP to Enhanced community treatment in patients with GAD or Anxiety NOS.	MP would improve anxiety, depression, and quality of life to a greater degree than Enhanced community treatment.	C F2F Indv	GAD or Anx NOS	1. MP (15) 2. Enchanced community treatment (16)	1: 71 (7) 2: 73,3 (6,3)	83,9	GAD (N = 27) Anx NOS (N = 4)	Yes	Yes	No
Wuthrich & Rapee, 2013 [81]	AU	To compare CBT to a WLC in older patients with comorbid anxiety and depression.	CBT would produce significant improvements on all symptom measures at post-treatment. Improvements would be maintained at the 3-month follow-up.	C F2F Group	Anxiety + depression symptoms	1. CBT (27) 2. WLC (35)	67,44 (6,19)	64,5	GAD (N = 21) SAD (N = 6) SP (N = 1) PTSD (N = 3) Dep (N = 29) Anx NOS (N = 2)	Yes	Yes	No
Wuthrich et al. 2016 [53]	AU	To compare CBT to a discussion group in older patients with comorbid anxiety and depression.	Both conditions would improve diagnostic severity and symptom outcomes. CBT would improve anxiety and depression and diagnostic severity to a greater degree than the discussion group Improvements of participants allocated to CBT would be maintained at the 6-month follow-up.	C F2F Group	Anxiety disorder + depressive disorder	1. CBT (76) 2. Discussion group (57)	67,35 (5,44)	55,6	GAD (N = 44) Dep (N = 37)	No	No	No

*Note*. Ctry: Country; Comorb: Comorbidity; C: Community; F2F: Face-to-face; Indv: Individual; N/A: Not available; SP: Specialized care; PC: Primary care; T: Telephone; Univ stud: University students; Comp: Computerized; PD/AG: Panic disorder/agoraphobia; SAD: Social anxiety disorder; SP: Specific phobia; OCD: Obsessive-compulsive disorder; GAD: Generalized anxiety disorder; PTSD: Posttraumatic stress disorder; Anx NOS: Anxiety disorder not otherwise specified; MDD: Major depressive disorder; Dep NOS: Depressive disorder not otherwise specified; Dep: Depression (major depressive disorder, dysthymic disorder or dep NOS); M anx-dep: Mixed anxiety and depression; ACT: Acceptance and Commitment Therapy; CBT: Cognitive Behavioral Therapy; MBSR: Mindfulness-based Stress Reduction; UP: Unified Protocol; SD-CBT: Single-disorder Cognitive Behavioral Therapy; WLC: Waiting-list Control; SC: Supportive Counseling; T-CBT: Tailored Cognitive Behavioral Therapy; CAU: Care as Usual; MT: Mindfulness Treatment; CBT-T: Cognitive Behavioral Therapy delivered by Telephone; IO: Information-only; STPP: Short-term Psychodynamic Psychotherapy; TAU: Treatment as Usual; AC: Attention Control; MMI: Multimodal Intervention; PP: Psychodynamic Psychotherapy; MBCT: Mindfulness-based Cognitive Therapy; P-CT: Present-centered Therapy; SE: Self-exposure; DBT: Dialectical Behavioral Therapy; FSBET: False Safety Behavior Elimination Therapy; CAST: Cognitive Anxiety Sensitivity Treatment; CBM: Cognitive Bias Modification; PHET: Physical Health Education Training; PAI: Positive Activity Intervention; MP: Modular Psychotherapy; Hp: Hypochondriasis; SD: Somatoform disorder; AUD: Alcohol use disorder; AD: Adjustment disorder; SUD: Substance use disorder

^a^Data on diagnoses from Bolton et al. (2014) [42] were not included in the analysis because patients with PTSD could not be distinguished from those with Dep (i.e. we could not determine whether patients had both PTSD and Dep, or how many patients had PTSD and how many had Dep)

^b^Data from Day et al. (2013) [60] were not included in the analysis because no information on diagnoses was provided in this study

### Quality of the included studies

**Fig 2:**
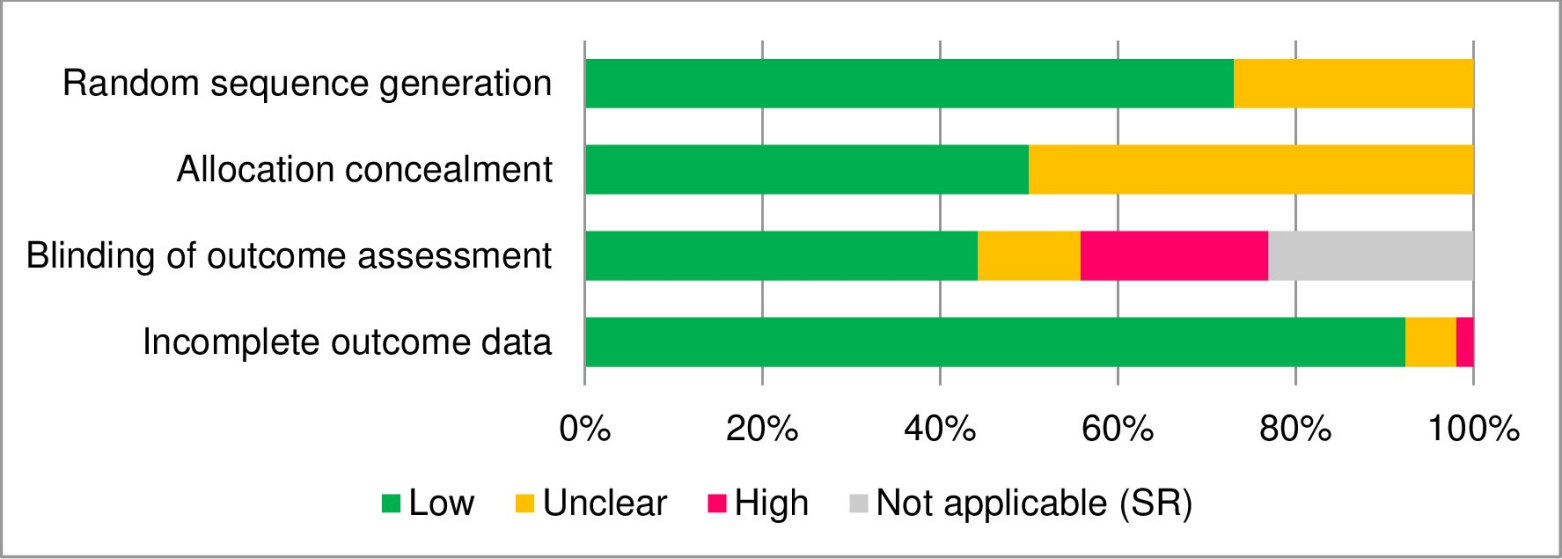
Risk of bias assessment. *Note*. SR = Self-report.

The risk of bias assessment of the included trials is represented in Fig 2. In all, 38 of the 52 studies (73%) used an adequate random sequence generation method, whereas 14 studies did not report information about the randomization method. Allocation concealment was reached in 26 of the assessed trials (50%), but it was not clearly reported in the other half (50%). With regard to blinding the outcome assessment, 23 trials (44%) reported using blinded raters, whereas 12 (23%) used only self-report measures. Almost all of the studies (92%) used an appropriate method for handling incomplete outcome data (i.e. intention-to-treat analyses). Sixteen studies (31%) met all the quality criteria, 30 studies (58%) met two or three criteria, and the six remaining trials met none or only one quality criterion.

### Are comorbid disorders evaluated in transdiagnostic treatments for emotional disorders?

We were also interested in the number of studies that reported and assessed comorbidity in their samples. Of the 52 studies analyzed, 39 (75%) reported the presence of comorbid disorders (i.e. whether the sample presented comorbidity at baseline), and 13 (25%) did not. However, of the total number of studies, only 21 (40%) assessed the effects of the intervention on comorbid disorders (i.e. through scales or diagnostic interviews).

### What diagnoses are targeted in transdiagnostic treatments for emotional disorders?

**Fig 3:**
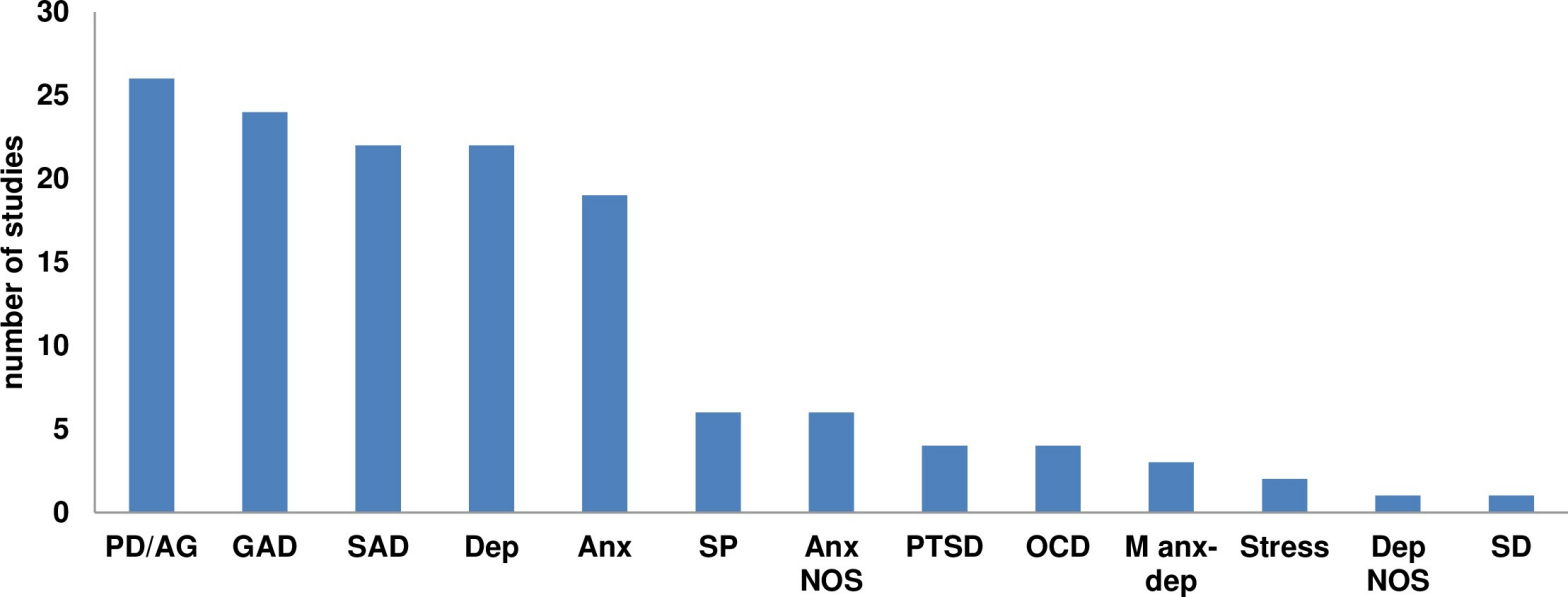
Number of studies that target the different diagnoses. *Note*. PD/AG: Panic disorder/agoraphobia; GAD: Generalized anxiety disorder; SAD: Social anxiety disorder; Dep: Depression; Anx: Anxiety; SP: Specific phobia; Anx NOS: Anxiety not otherwise specified; PTSD: Posttraumatic stress disorder; OCD: Obsessive-compulsive disorder; M anx-dep: mixed anxiety and depression disorder; Dep NOS: Depression not otherwise specified; SD: somatoform disorder.

Fig 3 presents the number of studies that target each of the different diagnoses. In this figure, both specific diagnoses and broad diagnosis categories (i.e. anxiety, depression, and mixed anxiety-depression) are shown because we identified studies that targeted either specific diagnoses or broader categories of anxiety and depression.

Of the 52 studies included in the review, the most commonly targeted diagnoses were PD/AG, (26 studies; 50%), GAD (24 studies; 46%), and SAD (22 studies; 42%). In addition, SP was targeted in 6 studies (12%) [31, 32, 62, 69, 76, 77], Anxiety NOS in 6 studies (12%) [20, 44, 46, 57, 58, 68], PTSD in 4 studies (8%) [32, 42, 47, 71], and OCD in 4 studies (8%) [18, 31, 32, 71]. Moreover, we identified 1 study targeting Depression NOS (2%) [46] and 1 study targeting somatoform disorders (2%) [70]. Finally, 22 studies targeted symptoms or diagnoses of depressive disorders (i.e. MDD or DD) (42%), 19 studies targeted symptoms or diagnoses of anxiety disorders (any type) (36.5%), 15 targeted depression symptoms or diagnoses (any type) (29%), 3 targeted mixed anxiety and depression (6%) [25, 52, 63], and 2 targeted stress (4%) [60, 70].

### What is the real distribution of diagnoses at baseline in transdiagnostic treatments for emotional disorders?

**Fig 4:**
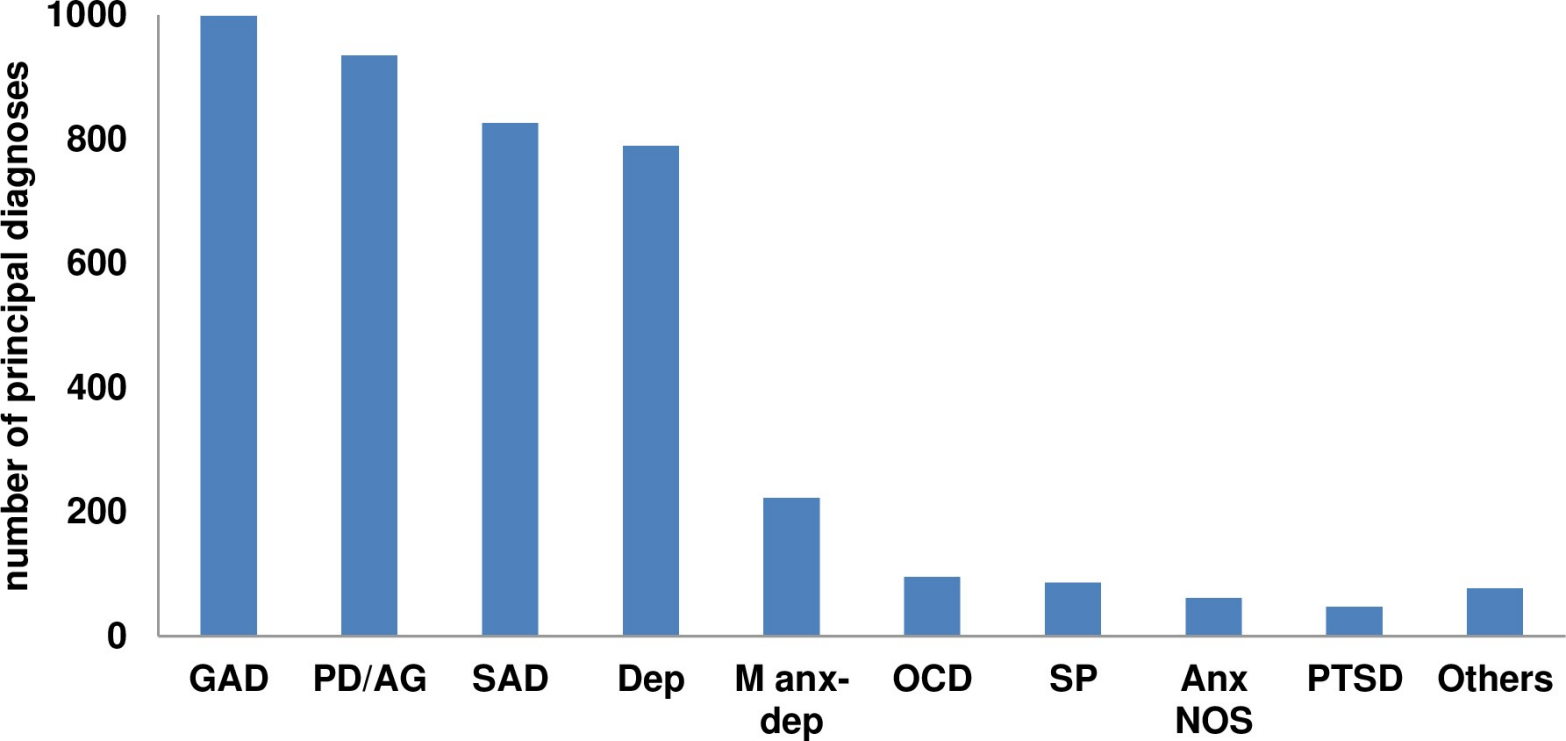
Total number of principal diagnoses of each type in subsample 1. *Note*. GAD: Generalized anxiety disorder; PD/AG: Panic disorder and/or agoraphobia; SAD: Social anxiety disorder; Dep: Depression; M anx-dep: Mixed anxiety and depression; OCD: Obsessive-compulsive disorder; SP: Specific phobia; Anx NOS: Anxiety not otherwise specified; PTSD: Posttraumatic stress disorder. “Others” included GAD + MDD (n = 47), GAD + PD (n = 25), GAD + SAD (n = 1), SAD + Anxiety NOS (n = 1), OCD + PD/AG (n = 1), and anxiety/depression NOS (n = 2).

In order to obtain the distribution of each of the different diagnoses, we classified the studies into those that reported a principal diagnosis (subsample 1) and those that did not (subsample 2). Of the 52 studies included in the review, 36 established a principal diagnosis, and 4125 patients with a principal diagnosis were identified in this subsample. The proportion of these patients for each of the different principal diagnoses can be seen in Fig 4. The most common diagnoses were GAD (n = 998; 24.1%), PD/AG (n = 935; 22.6%), SAD (n = 826; 20.0%), Dep (i.e. MDD or DD) (n = 789; 19.1%), and mixed anxiety and depression (n = 222; 5.4%). Other much less frequent diagnoses were OCD (n = 95, 2.3.%), SP (n = 86; 2.1%), Anxiety NOS (n = 61; 1.5%), and PTSD (n = 47; 1.1%).

**Fig 5:**
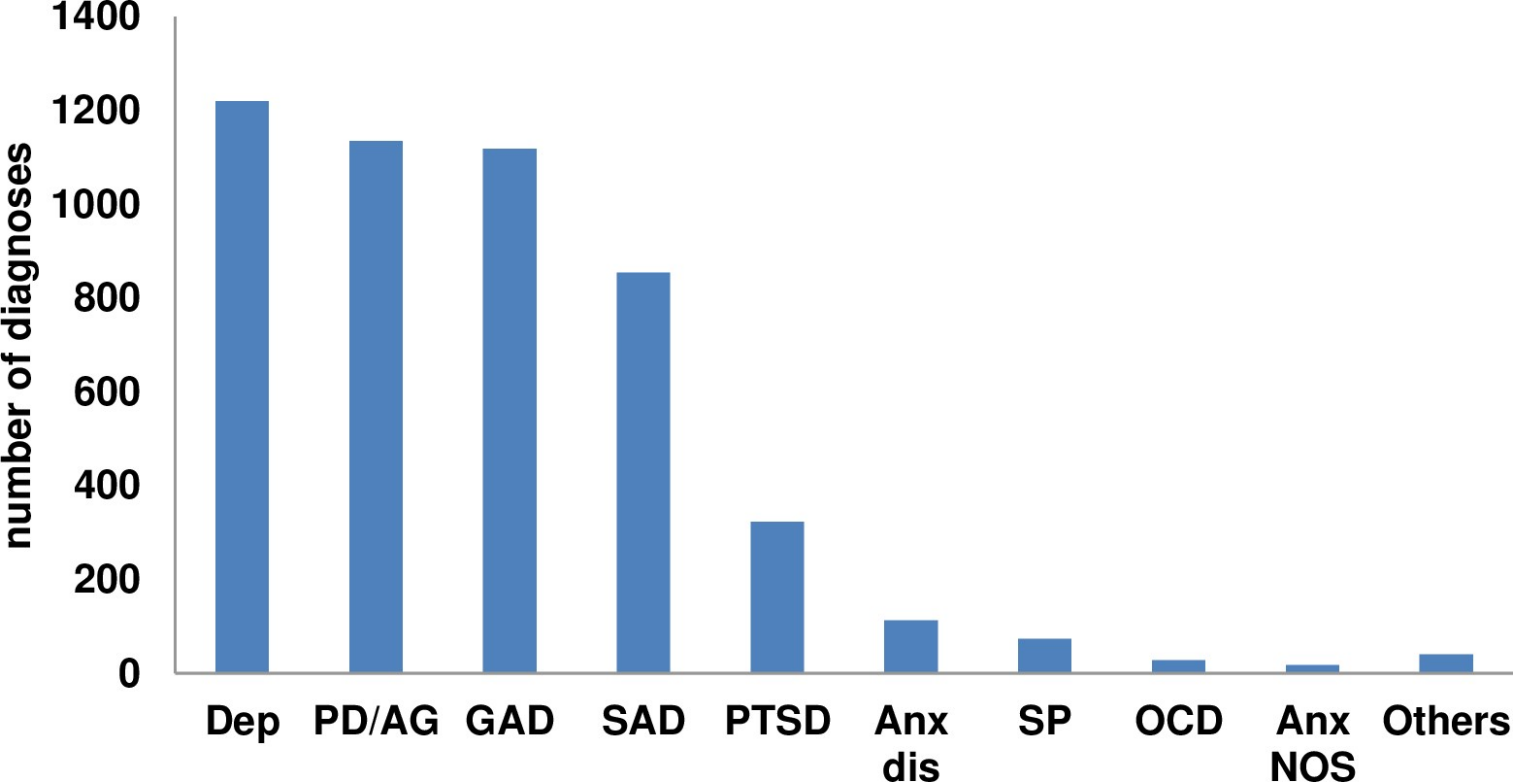
Total number of diagnoses of each type in subsample 2. *Note*. Dep: Depression; PD/AG: Panic disorder and/or agoraphobia; GAD: Generalized anxiety disorder; SAD: Social anxiety disorder; PTSD: Posttraumatic stress disorder; Anx dis: Anxiety disorders; SP: Specific phobia; OCD: Obsessive-compulsive disorder; Anx NOS: Anxiety not otherwise specified. “Others” included somatoform disorder (n = 10), adjustment disorder (n = 10), eating disorders (n = 9), alcohol use disorder (n = 4), substance use disorder (n = 4), and anxiety/depression NOS (n = 4).

The proportion of different diagnoses in the studies that did not include information about a principal diagnosis (subsample 2) is shown in Fig 5. In this subsample, a total of 4926 diagnoses were identified (pertaining to 2882 patients), and the most common diagnoses were Dep (n = 1220; 24.8%), PD/AG (n = 1135; 23%), GAD (n = 1119; 22.7%), SAD (n = 855; 17.4%), and PTSD (n = 323; 6.6%). Other disorders in these studies included SP (n = 74; 1.5%), OCD (n = 28; 0.6%), and Anxiety NOS (n = 18; 0.4%).

## Discussion

The aim of this systematic review was to analyze the following aspects about transdiagnostic treatments for ED: first, whether treatment response in the comorbid disorders is evaluated in transdiagnostic treatments for ED; second, what disorders are targeted in these studies; and third, what the real distribution of these disorders is at baseline in these studies.

The first objective was to analyze how comorbidity is reported and whether the treatment change produced in comorbid disorders is assessed in transdiagnostic trials for ED. The results showed that the number of studies reporting comorbidity was quite high, with 39 out of 52 reporting the presence of comorbid disorders in their samples at baseline. However, the number of studies assessing comorbidity was much lower, with only 21 (40.4%) studies assessing the comorbid conditions as well as the symptoms of the principal diagnosis, using either diagnostic interviews [18, 22] or self-report questionnaires [21, 40, 41]. From a transdiagnostic perspective that addresses the common maintenance vulnerabilities across disorders (e.g. neuroticism), it makes more sense to explore the extent to which these treatments are effective in improving both principal and comorbid disorders. In order to gain insight into how transdiagnostic treatments work in patients with comorbidity, we believe this strategy should be followed in future research on transdiagnostic treatments for ED. Furthermore, future meta-analyses of transdiagnostic treatments for ED would benefit from this strategy because they could analyze the impact of these treatments on comorbidities, in addition to their effects on broader measures of anxiety and depression. To date, some studies have included measures to assess treatment response in comorbidity in addition to the principal diagnosis [e.g. 21–23]. However, as the results of this systematic review show, this is not the typical approach in RCTs on transdiagnostic treatments (i.e. only 40.4% of the trials analyzed in this review assessed the impact of the intervention on comorbidity). As an example of this emphasis on comorbid diagnoses, a recent study tested the efficacy of a transdiagnostic treatment (the UP), compared to disorder-specific CBT with a specific focus on comorbid conditions, finding no differences in efficacy between the two treatment approaches [82]. These authors have also acknowledged the low number of treatments that, in general, evaluate treatment effects on comorbid disorders [82], whereas other authors have highlighted that transdiagnostic treatments should improve not only overall anxiety and depression, but also the disorder-specific comorbid psychopathology [38]. In this vein, most of the meta-analyses published to date have only analyzed the effects of transdiagnostic treatments using measures of overall anxiety and depression, except one recent meta-analysis that also explored the impact of these interventions on comorbidities [38]. To do so, the authors compared the effects of transdiagnostic treatments for ED to control conditions on disorder-specific measures of generalized anxiety, panic, and social anxiety. However, only 5 studies were included in this meta-analysis, which suggests the overall lack of attention paid to the evalution of comorbidity in transdiagnostic treatments.

It is important to note that some of the studies included in this review follow a treatment perspective that does not fall into the “shared mechanisms approach” described by Sauer-Zavala et al. [13]. Some of these approaches include tailoring the treatment to the specific disorders and comorbidities of each individual [19, 20, 73], changing the relationship of the patient with her or his own subjective experience (regardless of the specific disorder involved) through the delivery of “third wave” therapies (e.g. mindfulness, acceptance, and commitment therapy) [31, 32, 43, 51], or helping the patients to resolve their inner psychic conflicts using psychodynamic therapy [46, 69]. Regarding the usefulness of these transdiagnostic treatments for comorbid presentations, whereas tailored treatments aim to tailor the treatment according to the specific symptoms of the patient, third wave and psychodynamic therapies are considered transdiagnostic because they are usually applied indistinctly to treat different types of disorders. In sum, all of these approaches represent different strategies used to target comorbidity.

Regarding the second objective, (i.e. *what diagnoses are targeted in transdiagnostic treatments for emotional disorders*?*)* PD/AG was targeted in half of the studies, followed by GAD, SAD, and Dep, which were also targeted in almost half of the studies (46, 42, and 42%, respectively). By contrast, Anxiety NOS and Depression NOS were only targeted in 12 and 2% of the studies, respectively. Finally, the third question tried to answer *what the real distribution of the diagnoses is at baseline in transdiagnostic treatments for emotional disorders*. Regarding this question, the findings show that, in patients with a principal diagnosis (subsample 1), GAD was the most frequent diagnosis, followed by PD/AG, SAD, and Dep. Taken together, these disorders represented 85.8% of subsample 1, with anxiety disorders being the most common disorders targeted in transdiagnostic treatments for ED. Other ED appeared much less frequently. These disorders included OCD, SP, Anxiety NOS, PTSD, and Depression NOS. In patients with unreported principal diagnoses (subsample 2), Dep was the most common diagnosis, followed by PD/AG, GAD, SAD, and PTSD. These disorders represented 94.5% of the total number of diagnoses, and anxiety disorders were again the most frequent disorders targeted in the transdiagnostic treatments. By contrast, Anxiety NOS and OCD only represented 1% of this subsample. Overall (both subsamples), the most common disorders targeted in transdiagnostic trials were GAD, PD/AG, SAD, and Dep. These results are consistent with the high prevalence rates observed for these disorders [8, 9, 54]. For instance, according to the DSM-IV-TR [54], lifetime prevalence is 5% for GAD, 10–25% (female) and 5–12% (male) for major depression, 6% for dysthymic disorder, 1.5–3.5% for PD/AG, 2.5% for OCD, 3–13% for SAD, and 1–14% for PTSD. However, other ED, such as OCD, PTSD, Anxiety NOS, and SP, have received much less attention in the research on transdiagnostic treatments for ED, and they are usually not targeted in these protocols. On the one hand, it is worth noting that there is a low proportion of patients with OCD as the principal diagnosis included in the transdiagnostic interventions, even though this disorder can be appropriately treated from a transdiagnostic perspective, based on common maintenance vulnerabilities across ED [15, 17, 22]. In the case of PTSD, earlier studies with transdiagnostic protocols like the UP [83], which originally targeted this diagnosis, do not include this category in later studies [18, 22], in spite of the fact that this disorder might be an appropriate treatment target from a mechanistically transdiagnostic approach (i.e. a treatment approach that addresses the common underlying mechanisms across a range of disorders) [18, 84]. On the other hand, transdiagnostic treatments have the potential to target diagnoses that do not fit any specific category (e.g. Anxiety NOS) [12,15]. Although there are data indicating that there is a high proportion of these presentations [85, 86], the number of diagnoses with Anxiety NOS analyzed in this study represented less than 1% of all the patients. In this regard, one somewhat surprising result is that the overall number of patients with a diagnosis of SP is larger than the number of patients with Depression or Anxiety NOS, even though one of the advantages of the transdiagnostic perspective is the possibility of treating NOS diagnoses, clinical presentations for which there is a lack of evidence-based treatments.

Regarding the control conditions, both the waitlist control and the active control conditions were the most frequent among the analyzed studies. Of the studies that used active control conditions, only 8 were disorder-specific treatments. In order to accumulate evidence about the efficacy of transdiagnostic treatments, more studies should compare these protocols to disorder-specific treatments [21, 23]. Thus, although there is some evidence showing that a transdiagnostic approach may benefit depressive symptomatology more than disorder-specific protocols [36], overall the literature suggests that these two treatment approaches have equivalent effects [18, 39–41]. However, the number of studies comparing these two approaches is still low, and so more research is warranted to more firmly establish their relative efficacy. Likewise, research comparing the cost-effectiveness of transdiagnostic treatments and disorder-specific protocols is of paramount importance, for a number of reasons. First and foremost, by using a transdiagnostic treatment, less training of clinicians is required because a single protocol is used to address multiple disorders, which is likely to facilitate its implementation in real-world settings (e.g. primary care and mental health services). Second, these treatments may be more useful for clinicians that have to address comorbid presentations, either by targeting the underlying common processes, by tailoring the treatment to the symptoms and needs of each patient [20], or by addressing how the patients relate to their own cognitive, behavioral, and emotional experiences [31, 32]. Although the aforementioned reasons are true for most transdiagnostic treatments, there are other reasons specific to the protocols that fall in the category of the “shared mechanisms approach”. For instance, transdiagnostic treatments are designed to address the underlying common vulnerabilities across ED that are hypothesized to account for the onset and maintenance of these disorders [15]. Thus, by focusing on treating these processes rather than disorder-specific variations, larger and more lasting effects on clinical outcomes would be expected [13]. These results would lead to a lower prevalence of ED, and therefore to a decreased need for treatments in the short and long term, resulting in increased cost-effectiveness. For these reasons, more research on the cost-effectiveness of transdiagnostic treatments is needed, especially in comparison with disorder-specific protocols, as evidenced by the scarcity of studies of this kind found in this review (e.g. the study by Nordgren et al.) [67]. Given the substantial burden of ED and the lack of resources to tackle these disorders, especially in public services, research on how to enhance the cost-effectiveness of psychological interventions should be a research priority. A characteristic example of a treatment strategy to further increase the efficiency of transdiagnostic protocols entails personalizing the treatment to a specific presentation, i.e. by selecting the treatment components that best fit the specific set of symptoms or “weaknesses” shown by each patient [87], thereby lowering the number of sessions required to successfully treat an individual’s symptoms.

Regarding the settings, 71% of the studies were conducted in community samples, whereas 20% were carried out in primary or specialized care, and only 4% with university students. Thus, community samples continue to be the setting of choice when conducting transdiagnostic trials for ED. Regarding the way these treatments were delivered, approximately half of the studies were face-to-face, whereas the other half were delivered through Information and Communication Technologies (web-based and computerized), and only one study was delivered by telephone. These results are not surprising because research on Internet interventions has increased enormously in recent years, and these interventions have been applied to different problems using a variety of treatment approaches [88]. As the field of Internet interventions advances, researchers are more likely to select this delivery format to explore new interventions. Finally, transdiagnostic treatments were mostly individual, with 68% of the studies conducted in an individual format and the rest in groups. These results are not surprising because most transdiagnostic treatments were originally developed to be applied individually, with some exceptions [89]. However, the potential of transdiagnostic treatments for improving the dissemination of empirically supported treatments (i.e. only one protocol is needed to address a range of psychological disorders) may be enhanced by modifying the way the treatments are delivered [90]. For example, Internet or group formats can be used to reach a larger number of people in need of psychological help [92, 93], especially in ecological settings where resources are generally scarcer, such as primary care or public mental health units [91, 93].

Finally, regarding the risk of bias assessment, the overall quality of the trials included was acceptable, especially regarding the handling of incomplete outcome data, with almost all the studies using an appropriate approach. However, it is worth noting that a large percentage of the studies did not properly report specific methodological aspects, such as the sequence random generation method and whether it was performed by an independent party, which led us to rate it as unclear. In order to improve the methodological quality of trials and reduce the different sources of bias, we encourage authors to follow guidelines for conducting and reporting on clinical trials, such as the CONSORT statement (Consolidated Standards of Reporting Trials) [94, 95] or the SPIRIT guidelines (Standard Protocol Items: Recommendations for Interventional Trials) [96, 97].

### Limitations

This systematic review has several limitations that should be mentioned. First, although a comprehensive search was conducted (4 different databases were used), some important studies might have not been identified. Moreover, studies written in languages other than English were excluded, which might have affected the representativity of the findings in this study. Second, the generalizability of the results is also limited by the fact that most of the studies included in this review were conducted in Western countries. Third, although aspects of the methodology were unreported or not clear in some studies, we did not contact the authors of these studies to obtain information that might have clarified these details. Thus, aspects of the study methods that were not clear were rated as unclear. However, based on our experience in conducting prior systematic reviews, we have observed that contacting the authors of these studies is often very difficult and, therefore, impractical. Fourth, as in any systematic review, this study is vulnerable to publication bias, and so some relevant unpublished studies might have been missed.

### Conclusions

In conclusion, this systematic review found that, although most of the studies reported the presence of comorbid disorders in their samples at baseline, less than half of them evaluated the effects of the intervention on the comorbid disorders. Patients with comorbid disorders normally exhibit greater rates of severity, disability, and chronicity. One main reason for using a transdiagnostic approach to the treatment of ED is better management of comorbidity. Therefore, efforts should be made to assess the impact of the intervention on the comorbid disorders, in addition to the principal diagnoses targeted in these studies. On the other hand, as the results showed, the most commonly targeted diagnoses in transdiagnostic treatments were PD/AG, GAD, SAD, and Dep. More research is needed with other diagnoses much less targeted in transdiagnostic treatments, such as PTSD, OCD, and anxiety/depression NOS, to further explore the potential of transdiagnostic treatments in treating these disorders.

## Supporting information

S1 Table PRISMA 2009 checklist. (DOCX)

## Abbreviations

ED: Emotional disorders
CBT: Cognitive behavioral therapy
RCT: Randomized controlled trial
DSM-IV-TR: Diagnostic and Statistical Manual of mental disorders, 4^th^ edition-text revision
MDD: Major depressive disorder
DD: Dysthymic disorder
NOS: Not otherwise specified
GAD: Generalized anxiety disorder
PD: Panic disorder
AG: Agoraphobia
SAD: Social anxiety disorder
PTSD: Posttraumatic stress disorder
OCD: Obsessive-compulsive disorder
SP: Specific phobia
DSM-5: Diagnostic and Statistical Manual of mental disorders, 5^th^ edition
PRISMA: Preferred Reporting Items for Systematic Reviews and Meta-Analyses
SR: Self-report
Mixed anx-dep: Mixed anxiety and depression disorder
SD: Somatoform disorder
Dep: Depression
CONSORT: Consolidated Standards of Reporting Trials
SPIRIT: Standard Protocol Items: Recommendations for Interventional Trials
